# Association of noninvasive respiratory support with mortality and intubation rates in acute respiratory failure: a systematic review and network meta-analysis

**DOI:** 10.1186/s40560-021-00539-7

**Published:** 2021-04-12

**Authors:** Hideto Yasuda, Hiromu Okano, Takuya Mayumi, Masaki Nakane, Nobuaki Shime

**Affiliations:** 1 Department of Emergency and Critical Care Medicine, Jichi Medical University Saitama Medical Center, 1-847, Amanuma-cho, Oomiya-ku, Saitama-shi, Saitama, 330-8503 Japan; 2 Department of Clinical Research Education and Training Unit, Keio University Hospital Clinical and Translational Research Center (CTR), 35, Shinanomachi, Shinjuku-ku, Tokyo, 160-8582 Japan; 3 Department of Critical and Emergency Medicine, National Hospital Organization Yokohama Medical Center, 2-60-3, Harajyuku, Totsuka-ku, Yokohama-shi, Kanagawa 245-8575 Japan; 4 Department of Cardiovascular Medicine, Graduate School of Medical Science, Kanazawa University, 1-13, Takaramachi, Kanazawa-shi, Ishikawa 920-0934 Japan; 5 Department of Emergency and Critical Care Medicine, Yamagata University Hospital, 2-2-2, Iidanishi, Yamagata-shi, Yamagata, 990-2331 Japan; 6 Department of Emergency and Critical Care Medicine, Postgraduate School of Medical Science, Hiroshima University Hospital, 3-2-1, Kasumi, Minami-ku, Hiroshima-shi, Hiroshima, 734-8551 Japan

**Keywords:** Acute hypoxic respiratory failure, Conventional oxygen therapy, Noninvasive ventilation, High-flow nasal cannula, Systematic review, Meta-analysis, Network meta-analysis

## Abstract

**Background:**

Noninvasive respiratory support devices may reduce the tracheal intubation rate compared with conventional oxygen therapy (COT). To date, few studies have compared high-flow nasal cannula (HFNC) use with noninvasive positive-pressure ventilation (NPPV). We conducted a network meta-analysis to compare the effectiveness of three respiratory support devices in patients with acute respiratory failure.

**Methods:**

The Cochrane Central Register of Controlled Trials, MEDLINE, EMBASE, and Ichushi databases were searched. Studies including adults aged ≥ 16 years with acute hypoxic respiratory failure and randomized-controlled trials that compared two different oxygenation devices (COT, NPPV, or HFNC) before tracheal intubation were included. A frequentist-based approach with a multivariate random-effects meta-analysis was used. The network meta-analysis was performed using the GRADE Working Group approach. The outcomes were short-term mortality and intubation rate.

**Results:**

Among 5507 records, 27 studies (4618 patients) were included. The main cause of acute hypoxic respiratory failure was pneumonia. Compared with COT, NPPV and HFNC use tended to reduce mortality (relative risk, 0.88 and 0.93, respectively; 95% confidence intervals, 0.76–1.01 and 0.80–1.08, respectively; both low certainty) and lower the risk of endotracheal intubation (0.81 and 0.78; 0.72–0.91 and 0.68–0.89, respectively; both low certainty); however, short-term mortality or intubation rates did not differ (0.94 and 1.04, respectively; 0.78–1.15 and 0.88–1.22, respectively; both low certainty) between NPPV and HFNC use.

**Conclusion:**

NPPV and HFNC use are associated with a decreased risk of endotracheal intubation; however, there are no significant differences in short-term mortality.

**Trial registration:**

PROSPERO (registration number: CRD42020139105, 01/21/2020)

**Supplementary Information:**

The online version contains supplementary material available at 10.1186/s40560-021-00539-7.

## Background

Acute respiratory failure (ARF) is prevalent among critically ill patients and is a common cause of intensive care unit (ICU) mortality [1, 2]. Approximately 60% of patients with ARF require invasive mechanical ventilation (IMV) [3], which is associated with adverse events, including ventilator-induced lung injury (VILI) and ventilator-associated pneumonia (VAP) [4, 5]. Patients with ARF on IMV have high hospital mortality rates of up to 30% [4]. Initial respiratory support, including conventional oxygen therapy (COT; e.g., nasal cannulas and facemasks), noninvasive positive-pressure ventilation (NPPV), and high-flow nasal cannula (HFNC) use, are important treatments to prevent tracheal intubation and reduce mortality among patients with hypoxic respiratory failure [6–10].

There is a widespread application of NPPV in patients with ARF before tracheal intubation and IMV [6–8] which decreases the need for IMV rather than the use of COT [11, 12]. NPPV potentially increases the risk of complications, including aspiration pneumonia, facial skin breakdown, eye irritation, interface intolerance, and patient discomfort from the inability to communicate or eat during therapy [13, 14], which limits NPPV application in the clinical setting. HFNC can deliver high-concentration humidified oxygen via nasal cannulas without NPPV-related complications and is increasingly used in critically ill adult patients despite contradictory results from several clinical trials [9, 10]. However, there is a paucity of evidence on pre-IMV HFNC use in patients with ARF.

Systematic reviews and meta-analyses that compared two of the three respiratory support devices (COT, NPPV, and HFNC) [15–22] showed that HFNC use reduced the tracheal intubation rate compared with COT, albeit without significance between-group differences when compared with NPPV. There was no intergroup difference in mortality between the use of any two of the three respiratory support devices. Several studies in those systematic reviews compared HFNC use with COT and NPPV with COT, although a few studies have compared HFNC use with NPPV. Small sample sizes possibly affected the results of the abovementioned systematic reviews. To overcome these limitations, we performed a systematic review and network meta-analysis (NMA) to compare the effectiveness of three supplemental respiratory support devices in studies that compared at least two of the three techniques (COT, NPPV, and HFNC use) in patients with ARF.

## Methods

### Protocol and registration

This systematic review was designed according to the Preferred Reporting Items for Systematic review and Meta-Analyses extension statement for reviews incorporating network meta-analyses (details in e-Table 1 in Additional file 1) [23], and the protocol is registered with PROSPERO (CRD42020139105).

### Eligibility criteria

#### Type of studies

We included all randomized-controlled trials (RCTs) reported in English and Japanese regardless of publication status (published, unpublished, and academic abstracts). Randomized crossover, cluster-randomized, and quasi-experimental trials were excluded.

#### Type of participants

This review included adults (age ≥ 16 years) with acute hypoxic respiratory failure, defined by any of the following criteria: ratio of arterial oxygen partial pressure to fractional inspired oxygen (P/F ratio) < 40.00 kPa; SaO_2_ or SpO_2_ < 94% on room air or > 95% with > 6 L/min; and PaO_2_ < 8.00 kPa with room air or < 10.67 kPa with O_2_. This meta-analysis excluded studies in which more than half of the patients had post-extubation respiratory failure, acute exacerbation of chronic obstructive pulmonary disease (COPD), acute exacerbation of asthma, hypercapnia (> 6.00 kPa), tracheostomy, post-surgical status, trauma, and do-not-resuscitate orders. The exclusion criteria were limited to factors that were judged clinically appropriate for exclusion by the participating clinicians.

#### Types of interventions and comparators

We included RCTs comparing two of the following three methods before tracheal intubation:

1. COT: Low-flow nasal cannula, face mask, and venturi mask (with no limit on the flow rate).
2. NPPV: The type of mask and mode, duration of ventilation, and methods of weaning were not limited.
3. HFNC: The flow rate and F_I_O_2_ were not limited.

#### Type of outcomes

The outcome measures included a primary outcome of short-term mortality at the end of the follow-up period (< 90 days), ICU discharge, and hospital discharge. The secondary outcome was the rate of intubation during ICU stay.

### Information sources

We searched for eligible trials in the following databases: The Cochrane Central Register of Controlled Trials (CENTRAL); MEDLINE via PubMed; EMBASE; and Ichushi, a database of Japanese research papers. Additionally, we searched for ongoing trials in The World Health Organization International Clinical Trials Platform Search Portal. For cases with unknown data, the authors were contacted.

### Search

We used the search terms “ARDS”, “adult respiratory distress syndrome”, “respiratory failure”, or “acute lung injury” AND “non-invasive ventilation”, “NPPV”, “oxygen therapy”, “HFNC”, or “high-flow therapy” in searches performed in December 2020 (details in e-Table 2 in Additional file 1).

### Study selection

Two of the three physicians (TM, HO, and HY) screened the title and abstract or the full text at the first and second screenings, respectively, for relevant studies and independently extracted data from the included studies into standardized data forms. Disagreements, if any, were resolved by discussion with one of three physicians who did not screen that particular study; original authors were contacted for clarification as required. For abstract-only studies that could not be evaluated for eligibility based on our review criteria, we attempted to contact the authors. Discrepancies between two reviewers were resolved by mutual discussion or discussion with a third reviewer as needed.

### Data collection process and data items

After identifying studies in the second screening, data were extracted from each study by the reviewers (TM, HO, and HY) using two tools: the Cochrane Data Collection Form (RCTs only) [24] and Review Manager (RevMan) software V.5.3.5 (Cochrane Collaboration) [25]. We extracted the following study characteristics:

1. Methods: study design, total study duration, number and locations of study centers, study setting, withdrawals, and date of study initiation
2. Participants: number, mean age, age range, sex, severity of condition, diagnostic criteria, and inclusion/exclusion criteria
3. Interventions: treatment approaches and comparison methods
4. Outcomes: primary and secondary outcomes that were specified and collected, and the timepoints reported

### Risk of bias within individual studies

The risk of bias of primary outcomes in the included studies was independently assessed by two of the three authors (TM and HO) using the Cochrane Risk of Bias tool 1.0 (Cochrane Collaboration) [26, 27] in seven domains: (a) random sequence generation, (b) allocation concealment, (c) blinding of participants and personnel, (d) blinding of outcome assessors, (e) incomplete outcome data, (f) selective outcome reporting, and (g) other sources of bias. The risk of each bias was graded as low, unclear, or high. Discrepancies between the two reviewers were resolved through discussion among themselves or with a third reviewer as necessary.

### Statistical analyses

#### Direct comparison meta-analysis

A pairwise meta-analysis was performed using RevMan 5.3 (RevMan 2014). Forest plots were used for meta-analysis, and the effect size was expressed as relative risk (RR) with the 95% confidence interval (CI) for categorical data and as weighted mean differences with the 95% CI for continuous data. Outcome measures were pooled using a random effect model for study-specific effects in measures. For all analyses, a two-sided *p* value < 0.05 was considered statistically significant.

Study heterogeneity between trials for each outcome was assessed by visually inspecting forest plots and with an *I*^2^ statistic to quantify inconsistency [28] (RevMan; *I*^2^ = 0–40, 30–60, 50–90, and 75–100% indicated minimal, moderate, substantial, and considerable heterogeneity, respectively). When heterogeneity was identified (*I*^2^ > 50%), we investigated the reason and quantified it using the Chi-square test.

We planned to use a funnel plot, Begg’s adjusted rank correlation test, and Egger’s regression asymmetry test to investigate publication bias if ≥ 10 studies were available (RevMan) [29]. As < 10 studies were included, we did not test for funnel plot asymmetry.

#### Network comparison meta-analysis

##### Data synthesis

A network plot was constructed to determine the number of studies and patients included in this meta-analysis. An NMA using netmeta 0.9-5 R-package (version 3.5.1) was performed via a frequentist-based approach with multivariate random-effects meta-analysis, and effect size was expressed as the RR (95% CI). Covariance between two estimates from the same study shows the variance of data in the shared arm, as calculated in a multivariable meta-analysis performed using the GRADE Working Group Approach for an NMA [30, 31].

##### Transitivity

The transitivity assumption underlying the NMA was evaluated by comparing the distribution of clinical and methodological variables that could act as effect modifiers across treatment comparisons.

##### Ranking

Ranking plots (rankograms) were constructed based on the probability that a given treatment had the highest event rate for each outcome. The surface under the cumulative ranking curve (SUCRA), which is a simple transformation of the mean rank, was used to determine treatment hierarchy [32] and was constructed using standard software (Stata 15.0, Stata, TX, USA).

##### Risk of bias across studies

The assessment of the risk of bias across studies followed considerations of pairwise meta-analysis, and conditions associated with “suspected” and “undetected” across-study bias were determined by the presence of publication bias on a direct comparison.

##### Indirectness

We evaluated the indirectness, classified as “no”, “some”, or “major” concern, of each study included in the NMA based on its relevance to the research question, including the study population, interventions, outcomes, and study setting. The study-level judgments could be combined with the percentage contribution matrix.

##### Imprecision

The approach to imprecision involved comparing the range of treatment effects included in the 95% CI with the range of equivalence. We assessed the heterogeneity of treatment effects for a clinically important risk ratio of < 0.8 or > 1.25 in the CIs.

##### Heterogeneity

To assess the level of heterogeneity, we compared the posterior and predictive distributions of the estimated heterogeneity variance [33]. Concordance between assessments based on CIs and prediction of intervals, both of which do not capture heterogeneity, were used to assess the importance of heterogeneity of treatment effects for a clinically important risk ratio of < 0.8 or > 1.25 in prediction intervals.

##### Assessment of inconsistency

The inconsistency of the network model was estimated using inconsistency factors and their uncertainties. We statistically evaluated consistency using the design-by-treatment interaction test [34]. For inconsistency, *p* values less than 0.05, between 0.05 and 0.10, and otherwise were classified as “Major concerns”, “Some concerns”, and “No concerns”, respectively.

#### Additional analyses

If there were sufficient data, we conducted a subgroup analysis of the severity of respiratory failure (P/F < 200) and the cause of respiratory failure (immunocompromised patients, excluding congestive heart failure (CHF)/acute exacerbation of COPD patients) to investigate the impact of risk of bias and assess the heterogeneity in participants in each study.

## Results

### Study selection

**Fig. 1:**
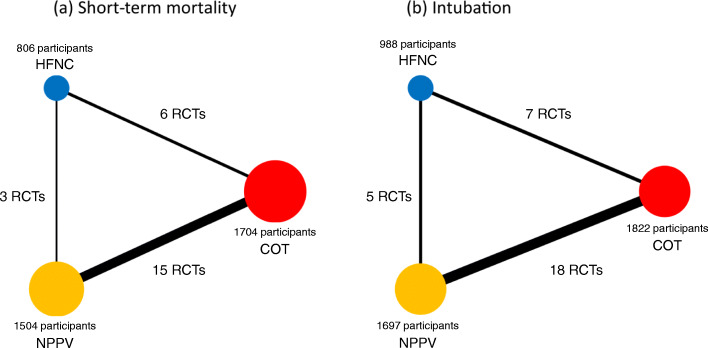
Network plots for the association of noninvasive oxygenation strategies with short-term mortality and intubation: a short-term mortality b Intubation

The comprehensive search yielded 5507 records (e-Fig 1 in Additional file 1), of which 27 studies were included in this NMA [9, 35–60]. These 27 studies included two three-group studies that directly compared NPPV with HFNC use and COT. The final analysis included 19, 7, and 5 studies that compared NPPV with COT, HFNC use with COT, and HFNC use with NPPV, respectively. The network structures of each outcome are shown in Fig. 1a and b.

### Study characteristics

The protocols and characteristics of each study included in the final dataset of the meta-analysis are summarized in Table 1. The quantitative analysis included 4618 patients. The main cause of acute hypoxic respiratory failure was pneumonia, followed by cardiopulmonary edema from CHF. Seven of the 27 included studies comprised many immunocompromised patients. The reported average P/F at randomization differed among the studies (range 95–249).

**Table 1:** Study populations, protocols, and characteristics

Author, year	Sample size *n*	Protocols			Baseline characteristics		
		Intervention setting	Control setting	Outcomes	The main causes of acute respiratory failure	Age, years	PaO_2_:F_I_O_2_
Bersten 1991 [51]	40	NPPV	COT	1. Mortality (in-hospital) 2. Intubation	CPE (CHF [56.4%])	NPPV: 76 (6) COT: 75 (6)	NPPV: 138 (32) COT: 136 (44)
Wysocki 1995 [36]	41	NPPV	COT	1. Mortality (in-ICU) 2.Intubation	AHRF (CHF [30%])	NPPV: 64 (18) COT: 62 (11)	NPPV: 191 (94) COT: 170 (77)
Antonelli 2000 [49]	40	NPPV	COT	1. Mortality (in-ICU) 2. Intubation	ARDS (patients who underwent solid organ transplantation [100%])	NPPV: 45 (19) COT: 44 (10)	NPPV: 142 (29) COT: 149 (22)
Delclaux 2000 [56]	123	NPPV	COT	1. Mortality (in-ICU, in-hospital) 2. Intubation	CPE (infection [49.6%])	NPPV: 56 [19–85]^a^ COT: 60 [18–88]^a^	NPPV: 140 [59–288]^a^ COT: 148 [62–283]^a^
Masip 2000 [39]	37	NPPV	COT	1. Intubation	CPE (myocardial infarction [29.7%])	NPPV: 75 (11) COT: 79 (5)	NA
Hilbert 2001 [46]	52	NPPV	COT	1. Mortality (in-ICU, in-hospital) 2. Intubation	CAP (immunocompromised patients [100%])	NPPV: 48 (14) COT: 50 (12)	NPPV: 141 (24) COT: 136 (23)
Levitt 2001 [41]	38	NPPV	COT	1. Intubation	CHF	NPPV: 67 (15) COT: 69 (15)	NA
Ferrer 2003 [54]	105	NPPV	COT	1. Mortality (in-ICU, 90 days) 2. Intubation	AHRF (Pneumonia [32.4%])	NPPV: 61 (17) COT: 62 (18)	NPPV: 102 (21) COT: 103 (23)
L’Her 2004 [44]	89	NPPV	COT	1. Mortality (48 h, in-hospital)	CPE (respiratory tract infection [33.7%])	NPPV: 84 (6) COT: 84 (6)	NPPV: 157 (71) COT: 167 (73)
Park 2004 [38]	80	NPPV	COT	1. Mortality (in-hospital, 15 days, 60 days) 2. Intubation	CPE (myocardial ischemia [37.5%])	NPPV: 64 (15) COT: 65 (15)	NA
Gray 2008 [53]	1069	NPPV	COT	1. Mortality (7 days, 30 days) 2. Intubation	CPE (ischemic heart disease [17.6%])	NPPV: 77 (10) COT: 79 (9)	NA
Cosentini 2010 [55]	47	NPPV	COT	1. Mortality (in-hospital) 2. Intubation	CAP [100%]	NPPV: 65 (17) COT: 72 (13)	NPPV: 249 (25) COT: 246 (20)
Qingyuan 2012 [35]	40	NPPV	COT	1.Moratlity (in-ICU, in-hospital) 2. Intubation	ALI (immunocompromised patients [30%])	NPPV: 44 (14) COT: 49 (14)	NPPV: 225 (17) COT: 234 (27)
Elena 2013 [37]	80	NPPV	COT	1. Intubation	AHRF (pneumonia [100%])	NA	NA
Brambilla 2014 [52]	81	NPPV	COT	1. Intubation	Pneumonia [100%]	NPPV: 65 (16) COT: 70 (16)	NPPV: 134 (32) COT: 148 (44)
Azevedo 2015 [47]	30	NPPV	HFNC	1. Intubation	AHRF (CHF [43%])	NA	NA
Frat 2015 [9]	313	NPPV/HFNC	COT	1. Mortality (in-ICU, 90 days) 2. Intubation	AHRF (CAP [62.9%])	NPPV: 61 (17) HFNC: 61 (16) COT: 59 (17)	NPPV: 149 (72) HFNC: 157 (89) COT: 161 (73)
Lemiale 2015 (1) [43]	374	NPPV	COT	1. Mortality (28 days) 2. Intubation	Pneumonia (immunocompromised patients [100%])	NPPV: 61 [52–70]^a^ COT: 64 [53–72]^a^	NPPV: 156 [95–248]^a^ COT: 130 [86–205]^a^
Lemiale 2015 (2) [42]	100	HFNC	COT	1. Intubation	AHRF (immunocompromised patients [100%])	HFNC: 59 [43–70]^a^ COT: 65 [53–72]^a^	NA
Frat 2016 [58]	86	NPPV/HFNC	COT	1. Mortality (in-ICU, 90 days) 2. Intubation	AHRF (immunocompromised patients [100%])	Total: 62 [48–74]	Total: 148 (58)
Jones 2016 [45]	303	HFNC	COT	1. Mortality (in-hospital) 2. Intubation	AHRF (COPD [23.9%])	HFNC: 75 (16) COT: 72 (17)	NA
Makdee 2017 [40]	128	HFNC	COT	1. Mortality (7 days) 2. Intubation	CPE	HFNC: 70 (16) COT: 71 (14)	NA
Azoulay 2018 [50]	778	HFNC	COT	1. Mortality (28 days) 2. Intubation	AHRF (immunocompromised patients [100%])	HFNC: 64 [55–70]^a^ COT: 63 [56–71]^a^	HFNC: 136 [96–187]^a^ COT: 128 [92–164]^a^
Doshi 2018 [57]	228	NPPV	HFNC	1. Intubation	AHRF (COPD exacerbation [26.0%])	NPPV: 63 (15) HFNC: 63 (14)	NA
Eman 2018 [48]	70	NPPV	HFNC	1. Mortality (in-hospital) 2. Intubation	AHRF (interstitial lung disease [100%])	NPPV: 61 (12) HFNC: 61 (12)	NPPV: 166 (42) HFNC: 178 (55)
Hangyong 2019 [59]	200	NPPV	COT	1. Mortality (in-hospital) 2. Intubation	AHRF (pneumonia [100%])	NPPV: 53 (18) COT: 56 (18)	NPPV: 232 (35) COT: 231 (28)
Andino 2020 [60]	46	HFNC	COT	1. Mortality (in-hospital) 2. Intubation	AHRF (pneumonia [62%])	HFNC: 58 (19) COT: 61 (11)	HFNC: 96 (29) COT: 95 (37)

*AHRF* Acute hypoxic respiratory failure; *ALI* Acute lung injury; *ARDS*, Acute respiratory distress syndrome; *CAP* Community-acquired pneumonia; *CHF* Congestive heart failure; *COPD* Chronic obstructive pulmonary disease; *COT* Conventional oxygen therapy; *CPE* Cardiogenic pulmonary edema; *HFNC* High-flow nasal cannula; *ICU* Intensive care unit; *NPPV* Noninvasive positive pressure ventilation

Continuous data were shown as mean and standard deviation, except for data labeled with “^a^”.

^a^ Data were reported as median and IQR (interquartile range).

### Risk of bias within studies

Additional file 1 e-Fig 2 shows the risk of bias; although all studies did not blind their participants and clinicians to the intervention, the risk of bias in the other domains was low (e-Fig 2 in Additional file 1). Finally, all included studies were judged as having low risks of bias for outcomes (risk of bias across studies).

### Network meta-analysis

The results of pairwise comparisons are shown in e-Fig 3 in Additional file 1 (short-term mortality) and e-Fig 4 in additional file 1 (Intubation). Additional file 1 e-Fig 5 shows the funnel plots of each outcome.

### Short-term mortality

**Fig. 2:**
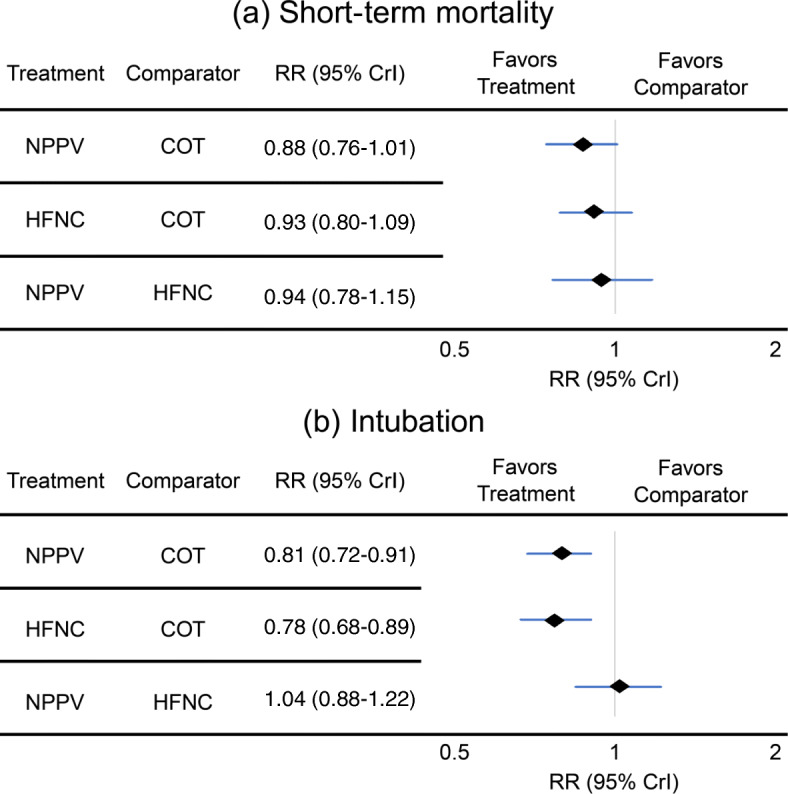
Forest plots of the network meta-analysis of the associations between noninvasive oxygenation strategies and short-term mortality and intubation. a Short-term mortality and b intubation

In the analysis of short-term mortality (including 20 studies), compared with COT, NPPV (RR, 0.88 [95% CI, 0.76–1.01]; low certainty) and HFNC use (RR, 0.32 [95% CI, 0.80–1.08]; low certainty) showed trends for lower mortality risk (Fig. 2a), and no significant difference was observed between NPPV and HFNC use for mortality (RR, 0.94 [95% CI, 0.78–1.15]; low certainty). Anticipated absolute effects and 95% CI between each of the two comparisons decreased by 28 per 1000 (95% CI, − 57 to + 2) in NPPV vs. COT, by 21 per 1000 (95% CI, − 61 to + 24) in HFNC use vs. COT, and by 9 per 1000 (95% CI, − 35 to + 24) in NPPV vs. HFNC use (Table 2).

**Table 2:**
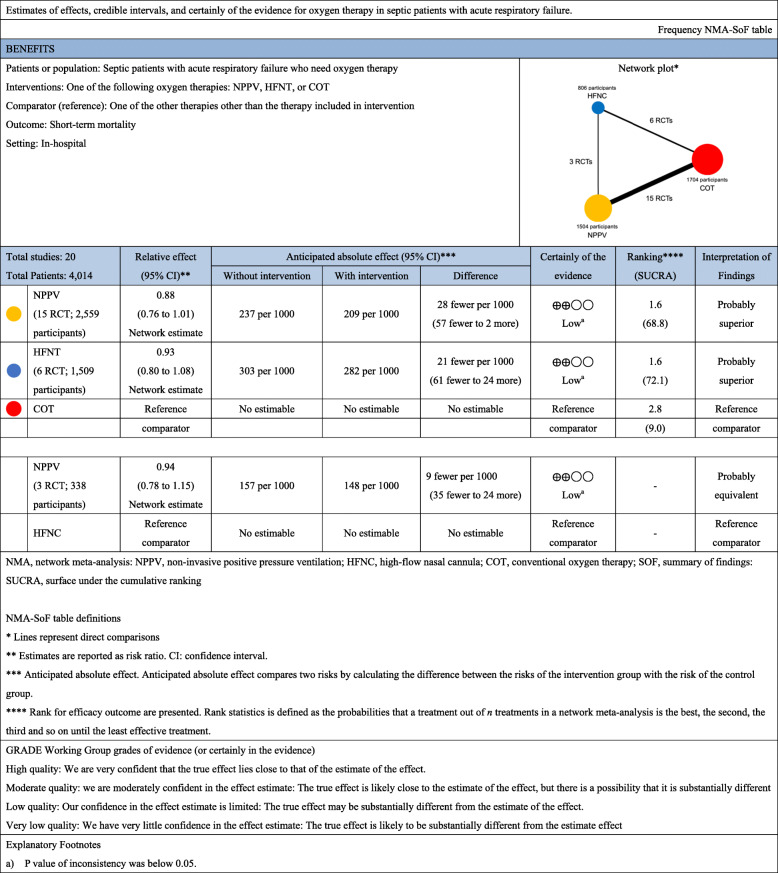
Summary of findings of network meta-analysis for short-term mortality

Confidence in the RR of each comparison and short-term mortality assessed by the GRADE system is shown in Table 3. Incoherence between direct and indirect RRs was observed for all three comparisons determined by *p* values of inconsistency. All comparisons (NPPV vs. COT, HFNC use vs. COT, and HFNC use vs. NPPV) showed “Major” concerns. The heterogeneity of all three comparisons resulted in “Major” concern outcomes due to the 95% CI of the predicted risk ratio.

**Table 3:** Confidence in the relative risk of each comparison and outcome assessed by the GRADE system for short-term mortality and intubation

	Risk of bias across studies	Imprecision	Heterogeneity	Indirectness	Publication bias	Incoherence	Confidence in relative risk of the event
**Short-term mortality**							
NPPV vs. COT	Undetected	Not serious (95% CI 0.76-1.01)	Major concern ^a)^ (95% PI 0.49-1.45)	Low	Not suggested	Major concern^b^ (*p* = 0.005)	⨁⨁◯◯ Low
HFNC vs. COT	Undetected	Not serious (95% CI 0.80-1.08)	Major concern ^a)^ (95% PI 0.45-1.47)	Low	Not suggested	Major concern^b^ (*p* = 0.031)	⨁⨁◯◯ Low
HFNC vs. NPPV	Undetected	Not serious (95% CI 0.78-1.15)	Major concern ^a)^ (95% PI 0.56-1.91)	Low	Not suggested	Major concern^b^ (*p* < 0.001)	⨁⨁◯◯ Low
**Intubation**							
NPPV vs. COT	Undetected	Not serious (95% CI 0.72-0.91)	Major concern ^a)^ (95% PI 0.29-1.46)	Low	Not suggested	Major concern^b^ (*p* = 0.0035)	⨁⨁◯◯ Low
HFNC vs. COT	Undetected	Not serious (95% CI 0.68-0.89)	Major concern ^a)^ (95% PI 0.26-1.44)	Low	Not suggested	Major concern^b^ (*p* = 0.001)	⨁⨁◯◯ Low
HFNC vs. NPPV	Undetected	Not serious (95% CI 0.88-1.22)	Major concern ^a)^ (95% PI 0.45-2.49)	Low	Not suggested	Major concern^b^ (*p* < 0.001)	⨁⨁◯◯ Low

*CI* Confidence interval; *COT* Conventional oxygen therapy; *HFNC* High-flow nasal therapy; *NPPV* noninvasive positive pressure ventilation; *PI* Prediction interval

^a^ *Prediction interval* ***extends*** *into clinically important effects in both directions.*

^b^ *P* value of inconsistency was below 0.05.

**Fig. 3:**
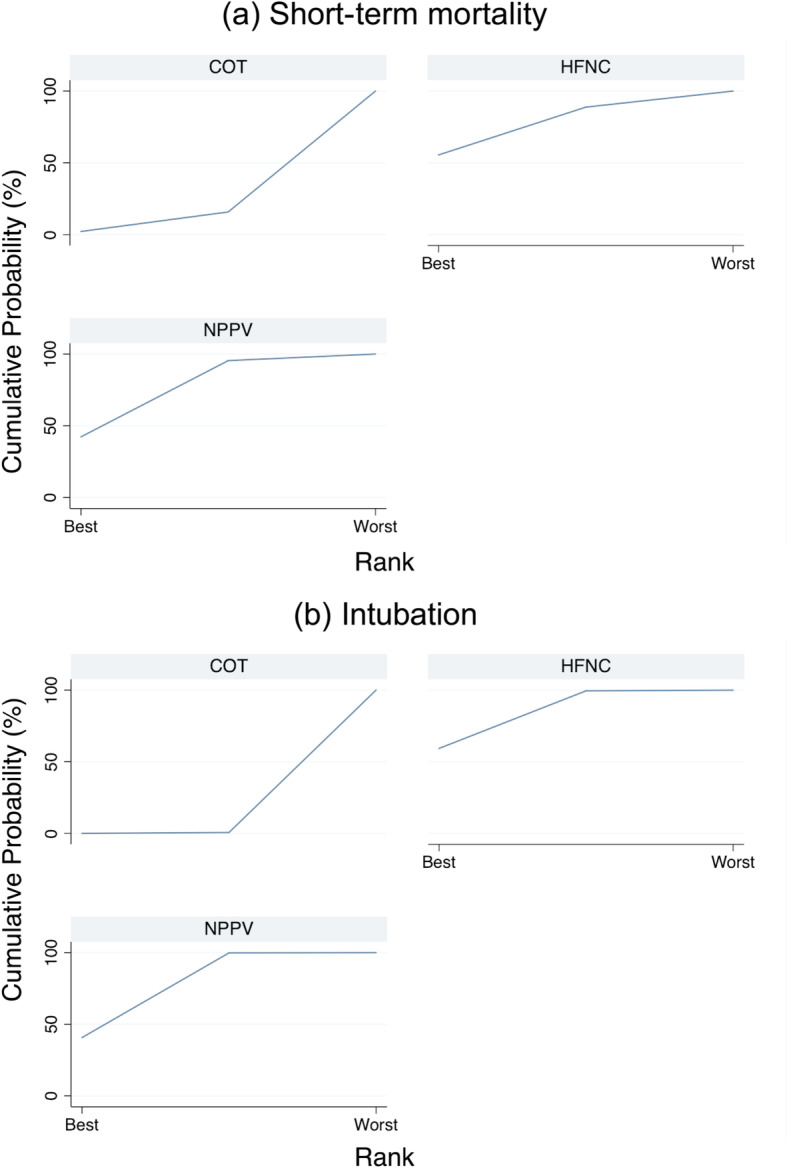
Surface under the cumulative ranking of each noninvasive oxygen strategies for short-term mortality and intubation: **a** short-term mortality, **b** intubation

A ranking analysis revealed that the hierarchy for efficacy in reducing short-term mortality was HFNC use (SUCRA 72.1), followed by NPPV (SUCRA 68.8) and ultimately, COT (SUCRA 9.0) (Fig. 3a). The summary of findings of the NMA for short-term mortality is shown in Table 2. The estimate and certainty of the evidence of direct, indirect, and network comparisons are summarized in e-Table 3 in additional file 1.

### Endotracheal intubation

Twenty-six studies were included in the analysis of endotracheal intubation. Compared with COT, NPPV (RR, 0.81 [95% CI, 0.72–0.91]; low certainty) and HFNC use (RR, 0.78 [95% CI, 0.68–0.89]; low certainty) were associated with statistically significant lower risks of endotracheal intubation (Fig. 2b), while no significant difference was observed between NPPV and HFNC use in the association with endotracheal intubation (RR, 1.04 [95% CI, 0.88–1.22]; low certainty). Anticipated absolute effects (95% CI) between each of the two comparisons decreased by 57 per 1000 (95% CI, − 83 to − 27) in NPPV vs. COT and 70 per 1000 (95% CI, − 101 to − 35) in HFNC use vs. COT, and increased by 9 per 1000 (95% CI, − 28 to + 51) in NPPV vs. HFNC use (Table 4).

**Table 4:**
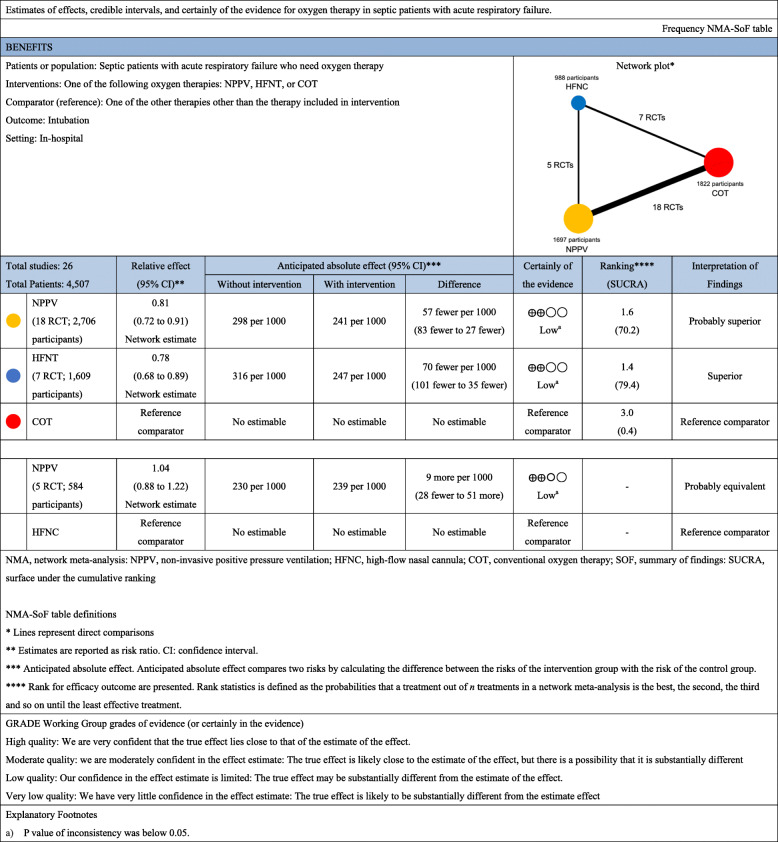
Summary of findings of network meta-analysis for intubation

Confidence in the RR of each comparison and intubation assessed according to the GRADE system (Table 3) showed incoherence between direct and indirect RRs for all three comparisons determined by the *p* value of inconsistency. All comparisons (NPPV vs. COT, HFNC use vs. COT, and HFNC use vs. NPPV) showed “Major” concerns. The heterogeneity of all three comparisons resulted in “Major” concern due to the 95% CI of the predicted risk ratio. The ranking analysis revealed that the hierarchy for efficacy in reducing intubation was HFNC use (SUCRA 79.4), followed by NPPV (SUCRA 70.2), and ultimately, COT (SUCRA 0.4) (Fig. 3b). Table 4 summarizes the NMA findings for intubation; e-Table 3 in additional file 1 summarizes the estimate and certainty of the evidence of direct, indirect, and network comparisons.

### Results of additional analyses

Of the 27 RCTs included in this study, only 14 focused on a single cause: 5, immunocompromised status; 4, pneumonia; 3, CHF; 1, post-transplant solid tumors; and 1, interstitial pneumonia. Therefore, the sensitivity analyses performed included the subgroup analysis of the severity of respiratory failure (P/F < 200) and the cause of respiratory failure (immunocompromised patients, excluding CHF/acute exacerbation of COPD patients). In all subgroup analyses, both short-term mortality and intubation rates were similar to those in the main analysis (e-Table 4 in Additional file 1).

## Discussion

This systematic review and NMA showed that NPPV and HFNC use were associated with lower risks of intubation compared with COT rather than an improved mortality risk. The SUCRA values of intubation for HFNC use and NPPV in the NMA showed similar effects to those for NPPV and HFNC use. These results are consistent with those of previous systematic reviews (non-NMA) [18, 21, 61]. A systematic review by Zhao et al. [21] that included 11 studies (*n* = 3459) compared HFNC use with COT or NPPV and found that unlike NPPV, HFNC use reduced the intubation rate compared with COT. Those studies differ from this study as they included many post-extubation studies. Despite the results of the previous review being consistent with those of our study—that HFNC use reduces the intubation rate compared with COT—the inclusion of studies examining the prevention of re-intubation after extubation would result in a deviation from the clinical question in this study. The sample size was inadequate due to the lack of RCTs that directly compared NPPV and HFNC use, and previous reviews have not shown a significant difference between NPPV and HFNC use. To complement the limitations of the existing studies, a systematic review using an NMA was necessary.

This NMA is the second study to demonstrate the effectiveness of NPPV and HFNC use in ARF. Ferreyro et al. [62] first reported an NMA describing the effects of noninvasive oxygenation strategies (e.g., NPPV and HFNC use) for patients with acute hypoxic respiratory failure. They concluded that treatment with noninvasive respiratory support devices was associated with a low mortality risk compared with standard oxygen therapy. Although the results of the intubation rate in this NMA are similar to those in the NMA by Ferreyro et al., the effect of NPPV and HFNC use on mortality, compared with that of COT, differed from the results in Ferreyro et al.’s study. In this NMA, we found no significant differences in the mortality risk between NPPV or HFNC use and COT.

Differences in the number of studies included in the NMA due to differences in the study inclusion criteria may have influenced the differences in the results of the two NMAs. First, this NMA included a large proportion of patients with CHF. Ferreyro et al. excluded studies in which patients with CHF constituted the majority of the study population. The clinical presentations of the cases of pneumonia and CHF are often complicated, with pneumonia being reported as a precipitating factor in CHF. Therefore, excluding studies that had patients with CHF may have affected the results of Ferreyro et al.’s NMA. Second, they included studies wherein patients with COPD constituted < 50% of the population while excluding studies that had a majority (> 50%) of patients with COPD. As noninvasive oxygenation strategies are useful in COPD [63], these studies with populations mostly consisting of patients with COPD may have influenced the NMA results. Additionally, the inclusion of patients with COPD increased the heterogeneity of the study population. Third, the NMA by Ferreyro et al. included the studies of ARF that occurred after abdominal surgery and chest trauma-associated respiratory failure, while excluding studies of patients after major cardiovascular surgery. Following abdominal surgery, diaphragmatic dysfunction and decreased vital lung capacity can cause atelectasis, resulting in hypoxemic respiratory failure (HRF). However, in pneumonia, which is the main cause of acute HRF, respiratory failure is caused by the decreased functional residual capacity due to inflammatory leachate in the alveoli and ventilator-perfusion mismatch. Analyzing these distinct pathogeneses of respiratory failure in a similar way is problematic and will affect the interpretation of results.

### Implications

For patients, respiratory management without intubation is obviously more comfortable. Despite the superiority of HFNC use and NPPV being inconclusive in our study, noninvasive respiratory management is useful because per 1000 patients, utilizing NPPV and HFNC will help avoid intubation in 57–70 patients compared with COT. IMV is associated with various adverse events (e.g., VILI and VAP) and needs specific skills for comfortable management. Respiratory management that avoids tracheal intubation can reduce such complications, and the patient is relieved of the intubation discomfort. For the hospital manager, the costs of respiratory management may decrease due to the lower rate of intubation as daily ventilation costs increase healthcare costs by 59% compared with non-ventilation costs [64].

### Limitations

This study had several limitations. First, there may be heterogeneity among the studies included, which may have affected the results. The NMA assumption is that the individual trials enrolled similar populations, and the intervention protocol was similar across different studies. Statistical heterogeneity is affected by a consequence of clinical or methodological diversity. Although statistical heterogeneity was a major concern in the mortality and intubation results, the clinical heterogeneity of diseases, including heart failure, pneumonia, and COPD, which were evaluated in this NMA, may not be considered high because of the difficulty in distinguishing between those diseases in the early stages of real-world clinical practice. The studies included in this NMA could not be clearly categorized by the cause of acute hypoxic respiratory failure because they did not include patients with a single cause. Therefore, sensitivity and subgroup analyses could not be performed.

Second, all RCTs included had high risks of performance bias due to the dramatic differences between HFNC use, COT, and NPPV, which made blinding impossible.

Third, although the statistical analysis was based on the assumption that there was no effect modifier, the inclusion of patients with various degrees of respiratory failure and different outcomes may have influenced the results due to undetectable effect modifiers. However, even for outcomes with different baseline risks, the relative effects of the interventions can remain consistent [65]. A sensitivity analysis based on respiratory failure severity should have been conducted; however, it was not performed because of the possibly similar severity (e.g., P/F ~ 200) of the patients in most studies included. Lastly, a few studies compared NPPV and HFNC use, and thus, the sample size was insufficient to compare NPPV and HFNC use. However, a trend in the direction of a difference is present, which may be further evaluated in the future if more studies compare these two noninvasive oxygenation strategies.

## Conclusions

The results of this NMA show that both NPPV and HFNC use are associated with lower risks of endotracheal intubation; however, no significant differences in short-term mortality exist between these respiratory support devices.

## Supplementary Information

**Additional file 1: e-Table 1.** PRISMA NMA Checklist of Items to Include When Reporting A Systematic Review Involving a Network Meta-analysis. **e-Table 2.** Search strategy. **e-Table 3.** Estimate and certainty of the evidence of direct, indirect, and network comparison: (a) Short-term mortality. (b) Intubation. **e-Table 4.** Summary of sensitivity analysis for the association of all interventions with outcomes: (a) Short-term mortality. (b) Intubation. **e-Fig 1.** PRISMA flow diagram (search, inclusion, and exclusion). **e-Fig 2.** Risk of bias summary for each comparison: (a) NPPV vs. COT. (b) HFNC vs. COT. (c) HFNC vs. NPPV. COT: conventional oxygen therapy, HFNC: high-flow nasal cannula oxygen, NPPV: noninvasive positive pressure ventilation. **e-Fig 3.** Forest plots for the pairwise comparison of short-term mortality: (a) NPPV vs. COT. (b) HFNC vs. COT. (c) HFNS vs. NPPV. COT: conventional oxygen therapy, HFNC: high-flow nasal cannula oxygen, NPPV: noninvasive positive pressure ventilation. **e-Fig 4.** Forest plots for the pairwise comparison of intubation: (a) NPPV vs. COT. (b) HFNC vs. COT. (c) HFNS vs. NPPV. COT: conventional oxygen therapy, HFNC: high-flow nasal cannula oxygen, NPPV: noninvasive positive pressure ventilation. **e-Fig 5.** Funnel plot of the comparison for NPPV and COT in each outcome: (a) Short-term mortality. (b) Intubation. COT: conventional oxygen therapy, NPPV: noninvasive positive pressure ventilation

## Data Availability

The datasets generated during and/or analyzed during the current study are not publicly available due to post hoc analyses by co-authors but are available from the corresponding author on reasonable request.

## Abbreviations

ARF: Acute respiratory failure
CENTRAL: Cochrane central register of controlled trials
CI: Confidence interval
COPD: Chronic obstructive pulmonary disease
COT: Conventional oxygen therapy
HFNC: High-flow nasal cannula
HRF: Hypoxemic respiratory failure
ICU: Intensive care unit
IMV: Invasive mechanical ventilation
NMA: Network meta-analysis
NPPV: Noninvasive positive-pressure ventilation
P/F ratio: Fractional inspired oxygen
RCT: Randomized controlled trial
RevMan: Review manager
RR: Relative risk
SUCRA: Surface under the cumulative ranking curve
VAP: Ventilator-associated pneumonia
VILI: Ventilator-induced lung injury
