# Supplementary material for: Association of noninvasive respiratory support with mortality and intubation rates in acute respiratory failure: a systematic review and network meta-analysis
Source: J Intensive Care. 2021 Apr 12;9:32. doi: 10.1186/s40560-021-00539-7 (PMC8041021; doi:10.1186/s40560-021-00539-7)
Supplement: Supplementary file 1 — Additional file 1: e-Table 1. PRISMA NMA Checklist of Items to Include When Reporting A Systematic Review Involving a Network Meta-analysis. e-Table 2. Search strategy. e-Table 3. Estimate and certainty of the evidence of direct, indirect, and network comparison: (a) Short-term mortality. (b) Intubation. e-Table 4. Summary of sensitivity analysis for the association of all interventions with outcomes: (a) Short-term mortality. (b) Intubation. e-Fig 1. PRISMA flow diagram (search, inclusion, and exclusion). e-Fig 2. Risk of bias summary for each comparison: (a) NPPV vs. COT. (b) HFNC vs. COT. (c) HFNC vs. NPPV. COT: conventional oxygen therapy, HFNC: high-flow nasal cannula oxygen, NPPV: noninvasive positive pressure ventilation. e-Fig 3. Forest plots for the pairwise comparison of short-term mortality: (a) NPPV vs. COT. (b) HFNC vs. COT. (c) HFNS vs. NPPV. COT: conventional oxygen therapy, HFNC: high-flow nasal cannula oxygen, NPPV: noninvasive positive pressure ventilation. e-Fig 4. Forest plots for the pairwise comparison of intubation: (a) NPPV vs. COT. (b) HFNC vs. COT. (c) HFNS vs. NPPV. COT: conventional oxygen therapy, HFNC: high-flow nasal cannula oxygen, NPPV: noninvasive positive pressure ventilation. e-Fig 5. Funnel plot of the comparison for NPPV and COT in each outcome: (a) Short-term mortality. (b) Intubation. COT: conventional oxygen therapy, NPPV: noninvasive positive pressure ventilation [file 40560_2021_539_MOESM1_ESM.docx]

**Additional file 1**

**Online-only tables**

**e-Table 1 PRISMA NMA Checklist of Items to Include When Reporting A Systematic Review Involving a Network Meta-analysis**

**e-Table 2 Search strategy**

**e-Table 3 Estimate and certainty of the evidence of direct, indirect, and network comparison: (a) Short-term mortality. (b) Intubation**

**e-Table 4 Summary of sensitivity analysis for the association of all interventions with outcomes: (a) Short-term mortality. (b) Intubation**

**Online-only Figs**

**e-Fig 1 PRISMA flow diagram (search, inclusion, and exclusion)**

**e-Fig 2 Risk of bias summary for each comparison: (a) NPPV vs. COT. (b) HFNC vs. COT. (c) HFNC vs. NPPV. COT: conventional oxygen therapy, HFNC: high-flow nasal cannula oxygen, NPPV: noninvasive positive pressure ventilation**

**e-Fig 3 Forest plots for the pairwise comparison of short-term mortality: (a) NPPV vs. COT. (b) HFNC vs. COT. (c) HFNS vs. NPPV. COT: conventional oxygen therapy, HFNC: high-flow nasal cannula oxygen, NPPV: noninvasive positive pressure ventilation**

**e-Fig 4 Forest plots for the pairwise comparison of intubation: (a) NPPV vs. COT. (b) HFNC vs. COT. (c) HFNS vs. NPPV. COT: conventional oxygen therapy, HFNC: high-flow nasal cannula oxygen, NPPV: noninvasive positive pressure ventilation**

**e-Fig 5 Funnel plot of the comparison for NPPV and COT in each outcome: (a) Short-term mortality. (b) Intubation. COT: conventional oxygen therapy, NPPV: noninvasive positive pressure ventilation**

**e-Table 1 PRISMA NMA Checklist of Items to Include When Reporting A Systematic Review Involving a Network Meta-analysis**

| **Section/Topic** | **Item #** | **Checklist Item** | **Reported on Page #** |
| --- | --- | --- | --- |
| **TITLE** |  |  |  |
| Title | 1 | Identify the report as a systematic review *incorporating a network meta-analysis (or related form of meta-analysis).* | 1 |
|  |  |  |  |
| **ABSTRACT** |  |  |  |
| Structured summary | 2 | Provide a structured summary including, as applicable:  **Background:** main objectives  **Methods:** data sources; study eligibility criteria, participants, and interventions; study appraisal; and *synthesis methods, such as network meta-analysis.*  **Results:** number of studies and participants identified; summary estimates with corresponding confidence/credible intervals; *treatment rankings may also be discussed. Authors may choose to summarize pairwise comparisons against a chosen treatment included in their analyses for brevity.*  **Discussion/Conclusions:** limitations; conclusions and implications of findings.  **Other:** primary source of funding; systematic review registration number with registry name. | 2 |
|  |  |  |  |
| **INTRODUCTION** |  |  |  |
| Rationale | 3 | Describe the rationale for the review in the context of what is already known*, including mention of why a network meta-analysis has been conducted.* | 3 |
| Objectives | 4 | Provide an explicit statement of questions being addressed, with reference to participants, interventions, comparisons, outcomes, and study design (PICOS). | 3 |
|  |  |  |  |
| **METHODS** |  |  |  |
| Protocol and registration | 5 | Indicate whether a review protocol exists and if and where it can be accessed (e.g., Web address); and, if available, provide registration information, including registration number. | 3 |
| Eligibility criteria | 6 | Specify study characteristics (e.g., PICOS, length of follow-up) and report characteristics (e.g., years considered, language, publication status) used as criteria for eligibility, giving rationale. *Clearly describe eligible treatments included in the treatment network, and note whether any have been clustered or merged into the same node (with justification).* | 4 |
| Information sources | 7 | Describe all information sources (e.g., databases with dates of coverage, contact with study authors to identify additional studies) in the search and date last searched. | 4 |
| Search | 8 | Present full electronic search strategy for at least one database, including any limits used, such that it could be repeated. | 4 |
| Study selection | 9 | State the process for selecting studies (i.e., screening, eligibility, included in systematic review, and, if applicable, included in the meta-analysis). | 5 |
| Data collection process | 10 | Describe method of data extraction from reports (e.g., piloted forms, independently, in duplicate) and any processes for obtaining and confirming data from investigators. | 5 |
| Data items | 11 | List and define all variables for which data were sought (e.g., PICOS, funding sources) and any assumptions and simplifications made. | 5 |
| **Geometry of the network** | **S1** | Describe methods used to explore the geometry of the treatment network under study and potential biases related to it. This should include how the evidence base has been graphically summarized for presentation, and what characteristics were compiled and used to describe the evidence base to readers. | 6 |
| Risk of bias within individual studies | 12 | Describe methods used for assessing risk of bias of individual studies (including specification of whether this was done at the study or outcome level), and how this information is to be used in any data synthesis. | 6 |
| Summary measures | 13 | State the principal summary measures (e.g., risk ratio, difference in means). *Also describe the use of additional summary measures assessed, such as treatment rankings and surface under the cumulative ranking curve (SUCRA) values, as well as modified approaches used to present summary findings from meta-analyses.* | 6 |
| Planned methods of analysis | 14 | Describe the methods of handling data and combining results of studies for each network meta-analysis. This should include, but not be limited to:   - *Handling of multi-arm trials;* - *Selection of variance structure;* - *Selection of prior distributions in Bayesian analyses; and* - *Assessment of model fit.* | 6 |
| **Assessment of Inconsistency** | **S2** | Describe the statistical methods used to evaluate the agreement of direct and indirect evidence in the treatment network(s) studied. Describe efforts taken to address its presence when found. | 7 |
| Risk of bias across studies | 15 | Specify any assessment of risk of bias that may affect the cumulative evidence (e.g., publication bias, selective reporting within studies). | 6 |
| Additional analyses | 16 | Describe methods of additional analyses if done, indicating which were pre-specified. This may include, but not be limited to, the following:   - Sensitivity or subgroup analyses; - Meta-regression analyses; - *Alternative formulations of the treatment network; and* - *Use of alternative prior distributions for Bayesian analyses (if applicable).* | 7 |
|  |  |  |  |
| **RESULTS†** |  |  |  |
| Study selection | 17 | Give numbers of studies screened, assessed for eligibility, and included in the review, with reasons for exclusions at each stage, ideally with a flow diagram. | 7 |
| **Presentation of network structure** | **S3** | Provide a network graph of the included studies to enable visualization of the geometry of the treatment network. | 8 |
| **Summary of network geometry** | **S4** | Provide a brief overview of characteristics of the treatment network. This may include commentary on the abundance of trials and randomized patients for the different interventions and pairwise comparisons in the network, gaps of evidence in the treatment network, and potential biases reflected by the network structure. | 7 |
| Study characteristics | 18 | For each study, present characteristics for which data were extracted (e.g., study size, PICOS, follow-up period) and provide the citations. | 7 |
| Risk of bias within studies | 19 | Present data on risk of bias of each study and, if available, any outcome level assessment. | 8 |
| Results of individual studies | 20 | For all outcomes considered (benefits or harms), present, for each study: 1) simple summary data for each intervention group, and 2) effect estimates and confidence intervals. *Modified approaches may be needed to deal with information from larger networks.* | 7 |
| Synthesis of results | 21 | Present results of each meta-analysis done, including confidence/credible intervals. *In larger networks, authors may focus on comparisons versus a particular comparator (e.g. placebo or standard care), with full findings presented in an appendix. League tables and forest plots may be considered to summarize pairwise comparisons.* If additional summary measures were explored (such as treatment rankings), these should also be presented. | 8-9 |
| **Exploration for inconsistency** | **S5** | Describe results from investigations of inconsistency. This may include such information as measures of model fit to compare consistency and inconsistency models, *P* values from statistical tests, or summary of inconsistency estimates from different parts of the treatment network. | 8-9 |
| Risk of bias across studies | 22 | Present results of any assessment of risk of bias across studies for the evidence base being studied. | 8-9 |
| Results of additional analyses | 23 | Give results of additional analyses, if done (e.g., sensitivity or subgroup analyses, meta-regression analyses*, alternative network geometries studied, alternative choice of prior distributions for Bayesian analyses,* and so forth). | 8-9 |
|  |  |  |  |
| **DISCUSSION** |  |  |  |
| Summary of evidence | 24 | Summarize the main findings, including the strength of evidence for each main outcome; consider their relevance to key groups (e.g., healthcare providers, users, and policy-makers). | 9 |
| Limitations | 25 | Discuss limitations at study and outcome level (e.g., risk of bias), and at review level (e.g., incomplete retrieval of identified research, reporting bias). *Comment on the validity of the assumptions, such as transitivity and consistency. Comment on any concerns regarding network geometry (e.g., avoidance of certain comparisons).* | 11 |
| Conclusions | 26 | Provide a general interpretation of the results in the context of other evidence, and implications for future research. | 11 |
|  |  |  |  |
| **FUNDING** |  |  |  |
| Funding | 27 | Describe sources of funding for the systematic review and other support (e.g., supply of data); role of funders for the systematic review. This should also include information regarding whether funding has been received from manufacturers of treatments in the network and/or whether some of the authors are content experts with professional conflicts of interest that could affect use of treatments in the network. | 12 |

PICOS = population, intervention, comparators, outcomes, study design.

* Text in italics indicateS wording specific to reporting of network meta-analyses that has been added to guidance from the PRISMA statement.

† Authors may wish to plan for use of appendices to present all relevant information in full detail for items in this section.

**e-Table 2 Search strategy**

1. **MEDLINE**

Performed on December 23^rd^ 2020

| Number | Searched for |
| --- | --- |
| #1 | "adult respiratory distress syndrome"[tiab] OR ”respiratory failure”[tiab] OR “Dyspnea”[tiab] OR “Dyspnea”[mesh] OR “shortness of breath “[tiab] OR "acute respiratory distress"[tiab] OR "Acute Respiratory Distress Syndrome" [tiab] OR "Respiratory Distress Syndrome, Adult" [mesh] |
| #2 | “Positive Pressure Respiration”[MeSH] OR “Noninvasive ventilation”[MeSH] OR “Noninvasive ventilation”[tiab] OR “Non invasive ventilation”[tiab] OR　“Noninvasive positive pressure ventilation”[tiab] OR “Non invasive positive pressure ventilation”[tiab] OR "NIPPV"[tiab] OR "NPPV"[tiab]　OR "NIV"[tiab] OR “Bilevel Positive Airway Pressure”[tiab] OR “Biphasic Positive Airway Pressure"[tiab] OR "BiPAP"[tiab] OR (“High Flow”[tiab] AND Nasal[tiab]) OR (“High Flow”[tiab] AND Therapy[tiab]) OR (“High Flow”[tiab] AND "Oxygen"[tiab]) OR　“HFNC”[tiab]　OR “HHFNC”[tiab] OR “HHHFNC”[tiab] OR “NHF”[tiab] |
| #3 | Randomized Controlled Trial[pt] OR Controlled Clinical Trial[pt] OR Randomized[tiab] OR Placebo[tiab] OR Drug Therapy[sh] OR Randomly[tiab] OR Trial[tiab] OR Groups[tiab] NOT (Animals [mh] NOT Humans [mh]) |
| #4 | #1 and #2 and #3 |

1. **CENTRAL**

Performed on December 23^rd^ 2020

| Number | Searched for |
| --- | --- |
| #1 | MeSH descriptor: [Respiratory Distress Syndrome, Adult] explode all trees |
| #2 | ("Acute Respiratory Distress Syndrome"):ti,ab,kw |
| #3 | ("acute respiratory distress"):ti,ab,kw |
| #4 | (”respiratory failure”):ti,ab,kw |
| #5 | (“shortness of breath “):ti,ab,kw |
| #6 | MeSH descriptor: [Dyspnea] explode all trees |
| #7 | (“Dyspnea”):ti,ab,kw |
| #8 | #1or #2 or #3 or #4 or #5 or #6 or #7 |
| #9 | MeSH descriptor: [Positive-Pressure Respiration] explode all trees |
| #10 | MeSH descriptor: [Noninvasive Ventilation] explode all trees |
| #11 | ("Noninvasive ventilation"):ti,ab,kw |
| #12 | ("Non invasive ventilation"):ti,ab,kw |
| #13 | ("Noninvasive positive pressure ventilation"):ti,ab,kw |
| #14 | ("Non invasive positive pressure ventilation"):ti,ab,kw |
| #15 | ("NIPPV"):ti,ab,kw |
| #16 | (“NPPV"):ti,ab,kw |
| #17 | ("NIV"):ti,ab,kw |
| #18 | ("Bilevel Positive Airway Pressure"):ti,ab,kw |
| #19 | “Biphasic Positive Airway Pressure” |
| #20 | ("BiPAP"):ti,ab,kw |
| #21 | MeSH descriptor: [Oxygen Inhalation Therapy] this term only |
| #22 | ("High Flow"):ti,ab,kw AND ("Nasal"):ti,ab,kw |
| #23 | (“High Flow"):ti,ab,kw AND ("Therapy"):ti,ab,kw |
| #24 | (“High Flow"):ti,ab,kw AND ("Oxygen"):ti,ab,kw |
| #25 | ("HFNC"):ti,ab,kw |
| #26 | (“HHFNC”):ti,ab,kw |
| #27 | (“HHHFNC”):ti,ab,kw |
| #28 | ("NHF"):ti,ab,kw |
| #29 | #10 or #11 or #12 or #13 or #14 or #15 or #16 or #17 or #18 or #19 or #20 or #21 #22 or #23 or #24 or #25 or #26 or #27 or #28 |
| #37 | #9 and #29 |

1. **EMBASE**

Performed on May 1^st^ 2019

| Number | Searched for |
| --- | --- |
| #1 | 'acute respiratory failure' |
| #2 | 'acute respiratory failure'/exp |
| #3 | 'shortness of breath' |
| #4 | 'adult respiratory distress syndrome' |
| #5 | 'acute respiratory distress syndrome' |
| #6 | 'adult respiratory distress syndrome'/exp |
| #7 | #1 OR #2 OR #3 OR #4 OR #5 OR #6 |
| #8 | 'noninvasive ventilation'/exp |
| #9 | 'noninvasive ventilation' |
| #10 | 'non invasive ventilation' |
| #11 | 'noninvasive positive pressure ventilation' |
| #12 | 'non invasive positive pressure ventilation' |
| #13 | nippv |
| #14 | nppv |
| #15 | niv |
| #16 | 'bilevel positive airway pressure' |
| #17 | 'biphasic positive airway pressure' |
| #18 | bipap |
| #19 | hfnc |
| #20 | hhfnc |
| #21 | hhhfnc |
| #22 | nhf* |
| #23 | #8 OR #9 OR #10 OR #11 OR #12 OR #13 OR #14 OR #15 OR #16 OR #17 OR #18 OR #19 OR #20 OR #21 OR #22 |
| #24 | 'high flow' AND therapy |
| #25 | 'high flow' AND oxygen* |
| #26 | 'high flow' AND nasal |
| #27 | #23 OR #24 OR #25 OR #26 |
| #28 | 'randomized controlled trial' |
| #29 | 'controlled clinical trial' |
| #30 | randomized |
| #31 | placebo |
| #32 | 'drug therapy' |
| #33 | randomly |
| #34 | trial |
| #35 | groups |
| #36 | #28 OR #29 OR #30 OR #31 OR #32 OR #33 OR #34 OR #35 |
| #37 | #7 AND #27 AND #36 |

1. **Ichushi**

Performed on December 23^rd^ 2020

| Number | Searched for |
| --- | --- |
| #1 | ((呼吸不全/TH or 呼吸不全/TA)) and (PT=会議録除く) |
| #2 | ((呼吸窮迫症候群-急性/TH or 呼吸窮迫症候群-急性/TA)) and (PT=会議録除く) |
| #3 | ((呼吸困難/TH or 呼吸困難/TA)) and (PT=会議録除く) |
| #4 | #1 or #2 or #3 |
| #5 | ((酸素吸入療法/TH or 酸素吸入療法/TA)) and (PT=会議録除く) |
| #6 | ((非侵襲的陽圧呼吸/TH or 非侵襲的陽圧換気/TA)) and (PT=会議録除く) |
| #7 | ((間欠的陽圧換気/TH or 間欠的陽圧換気/TA)) and (PT=会議録除く) |
| #8 | ((非侵襲的補助換気/TH or 非侵襲的補助換気/TA)) and (PT=会議録除く) |
| #9 | (陽圧呼吸/TH or 陽圧呼吸/TA) and (PT=会議録除く) |
| #10 | (持続気道陽圧/TH or 持続気道陽圧/TA) and (PT=会議録除く) |
| #11 | ((酸素マスク/TH or 酸素マスク/TA)) and (PT=会議録除く) |
| #12 | ((マスク/TH or マスク/TA)) and (PT=会議録除く) |
| #13 | (ベンチュリーマスク/TA) and (PT=会議録除く) |
| #14 | (HFNC/TA) and (PT=会議録除く) |
| #15 | (HFOT/TA) and (PT=会議録除く) |
| #16 | (高流量酸素療法/TA) and (PT=会議録除く) |
| #17 | (NIV/TA) and (PT=会議録除く) |
| #18 | (NPPV/TA) and (PT=会議録除く) |
| #19 | (NIPPV/TA) and (PT=会議録除く) |
| #20 | (NHFT/TA) and (PT=会議録除く) |
| #21 | #5 or #6 or #7 or #8 or #9 or #10 or #11 or #12 or #13 or #14 or #15 or #16 or #17 or #18 or #19 or #20 |
| #22 | ランダム化比較試験/TH or 準ランダム化比較試験/TH or ランダム化/AL or 無作為化/AL or 比較試験/AL or 臨床試験/AL or プラセボ/AL or 対照/AL or コントロール/AL or 臨床研究/AL |
| #23 | #4 and #21 and #22 |

**e-Table 3 Estimate and certainty of the evidence of direct, indirect, and network comparison: (a) Short-term mortality. (b) Intubation**

| 1. Short-term mortality | | | | | | |
| --- | --- | --- | --- | --- | --- | --- |
| Comparison | Estimate of direct comparison  (95% CI) | Certainly of the evidence of direct comparison | Estimate of indirect comparison  (95% CI) | Certainly of the evidence of indirect comparison | Estimate of network comparison  (95% CI) | Certainly of the evidence in network comparison |
| NPPV vs. COT | 0.81  (0.65,1.01) | ⨁⨁⨁◯ Moderate | 2.00  (1.11,3.62) | ⨁⨁⨁⨁ High | 0.88  (0.76,1.01) | ⨁⨁◯◯ Low^a)^ |
| HFNC vs. COT | 0.90  (0.66,1.24) | ⨁⨁⨁⨁ High | 0.45  (0.23,0.88) | ⨁⨁⨁◯ Moderate | 0.93  (0.80,1.08) | ⨁⨁◯◯ Low^a)^ |
| NPPV vs. HFNC | 1.90  (1.15,3.12) | ⨁⨁⨁⨁ High | 0.78  (0.62,0.97) | ⨁⨁⨁◯ Moderate | 0.94  (0.78,1.15) | ⨁⨁◯◯ Low^a)^ |
| 1. Intubation | | | | | | |
| NPPV vs. COT | 0.45  (0.45,0.80) | ⨁⨁⨁◯ Moderate | 1.22  (0.82,1.81) | ⨁⨁⨁⨁ High | 0.81  (0.72,0.91) | ⨁⨁〇◯ Low^a)^ |
| HFNC vs. COT | 0.84  (0.73,0.97) | ⨁⨁⨁⨁ High | 0.38  （0.24,0.58） | ⨁⨁⨁◯ Moderate | 0.78  (0.68,0.89) | ⨁⨁◯◯ Low^a)^ |
| NPPV vs. HFNC | 1.41  (1.09,1.82) | ⨁⨁⨁⨁ High | 0.82  (0.66,1.02) | ⨁⨁⨁◯ Moderate | 1.04  (0.88,1.22) | ⨁⨁〇◯ Low^a)^ |

CI, confidence interval; COT, conventional oxygen therapy; HFNC, high-flow nasal therapy; NPPV, non-invasive positive pressure ventilation

1. P value of inconsistency was below 0.05.

**e-Table 4 Summary of sensitivity analysis for the association of all interventions with outcomes: (a) Short-term mortality. (b) Intubation**

| 1. **Short-term mortality** |  |  |  |  |
| --- | --- | --- | --- | --- |
| Type of Analysis | No. of studies | NPPV vs. COT | HFNC vs. COT | NPPV vs. HFNC |
| All patients-Main analysis | 20 | 0.88 (0.76-1.01) | 0.93 (0.80-1.08) | 0.94 (0.78-1.15) |
| Immunocompromised patients | 4 | 0.84 (0.66-1.18) | 0.96 (0.81-1.13) | 0.88 (0.66-1.18) |
| Mean P/F Ratio < 200 | 13 | 0.88 (0.74-1.05) | 0.90 (0.77-1.05) | 0.98 (0.79-1.22) |
| Excluding studies with CHF/AECOPD patients | 11 | 0.93 (0.76-1.13) | 0.91 (0.78-1.06) | 1.02 (0.81-1.29) |
| 1. **Intubation** |  |  |  |  |
| All patients-Main analysis | 26 | 0.81 (0.72-0.91) | 0.78 (0.68-0.89) | 1.04 (0.88-1.22) |
| Immunocompromised patients | 5 | 0.91 (0.75-1.10) | 0.85 (0.73-1.00) | 1.07 (0.83-1.36) |
| Mean P/F Ratio < 200 | 13 | 0.85 (0.75-0.97) | 0.79 (0.69-0.91) | 1.07 (0.90-1.27) |
| Excluding studies with CHF/AECOPD patients | 14 | 0.85 (0.74-0.98) | 0.80 (0.69-0.92) | 1.07 (0.89-1.28) |

AECOPD, acute exacerbation of chronic obstructive pulmonary disease; CHF, congestive heart failure; COT, conservative oxygen therapy; HFNC, high-flow nasal cannula; NPPV, non-invasive positive pressure ventilation; P/F, PaO_2_/F_I_O_2_

**e-Fig 1 PRISMA flow diagram (search, inclusion, and exclusion)**

**e-Fig 2 Risk of bias summary for each comparison: (a) NPPV vs. COT. (b) HFNC vs. COT. (c) HFNC vs. NPPV. COT: conventional oxygen therapy, HFNC: high-flow nasal cannula oxygen, NPPV: noninvasive positive pressure ventilation**

**
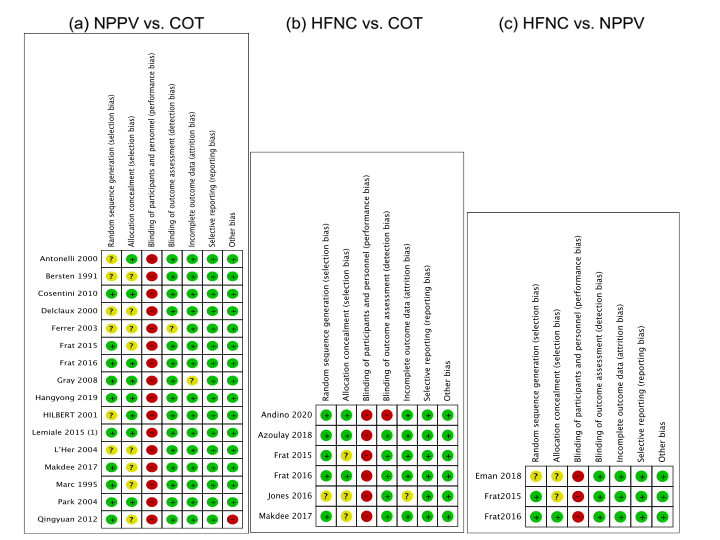
**

**e-Fig 3 Forest plots for the pairwise comparison of short-term mortality: (a) NPPV vs. COT. (b) HFNC vs. COT. (c) HFNS vs. NPPV. COT: conventional oxygen therapy, HFNC: high-flow nasal cannula oxygen, NPPV: noninvasive positive pressure ventilation**

**
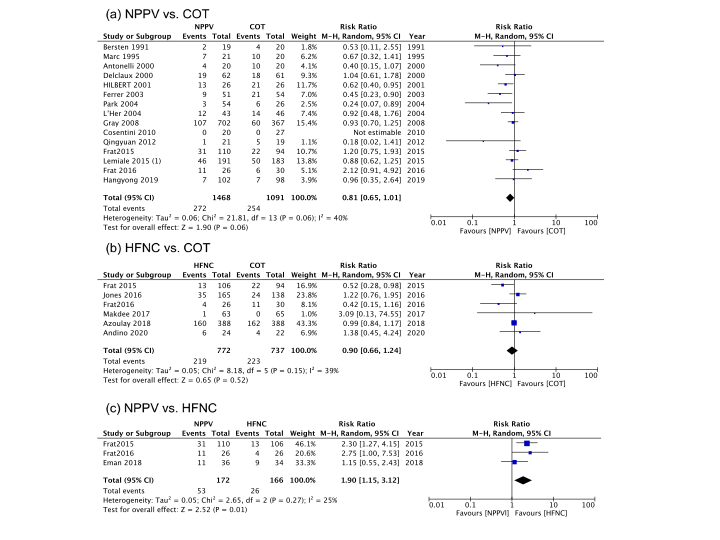
**

**e-Fig 4 Forest plots for the pairwise comparison of intubation: (a) NPPV vs. COT. (b) HFNC vs. COT. (c) HFNS vs. NPPV. COT: conventional oxygen therapy, HFNC: high-flow nasal cannula oxygen, NPPV: noninvasive positive pressure ventilation**

**
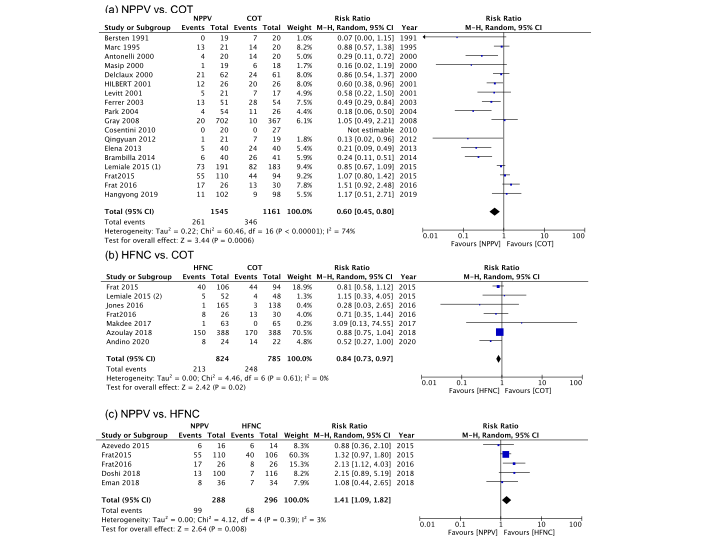
**

**e-Fig 5 Funnel plot of the comparison for NPPV and COT in each outcome: (a) Short-term mortality. (b) Intubation. COT: conventional oxygen therapy, NPPV: noninvasive positive pressure ventilation**

1. Short-term mortality

1. Intubation
